# Predicting the Neurodevelopmental Outcome in Extremely Preterm Newborns Using a Multimodal Prognostic Model Including Brain Function Information

**DOI:** 10.1001/jamanetworkopen.2023.1590

**Published:** 2023-03-08

**Authors:** Laura Routier, Laurent Querne, Ghida Ghostine-Ramadan, Julie Boulesteix, Solène Graïc, Sandrine Mony, Fabrice Wallois, Emilie Bourel-Ponchel

**Affiliations:** 1 INSERM UMR 1105, Research Group on Multimodal Analysis of Brain Function, University of Picardie Jules Verne, Amiens Cedex, France; 2 INSERM UMR 1105, Pediatric Neurophysiology Unit, Amiens-Picardie University Medical Center, Amiens Cedex, France; 3 Department of Pediatric Neurology, Amiens-Picardie University Medical Center, Amiens Cedex, France; 4 Neonatal Intensive Care Unit, Amiens-Picardie University Medical Center, Amiens Cedex, France

## Abstract

**Importance:**

Early assessment of the prognosis of preterm newborns is crucial for accurately informing parents and making treatment decisions. The currently available prognostic models rarely incorporate functional brain information from conventional electroencephalography (cEEG).

**Objective:**

To examine the performance of a multimodal model combining (1) brain function information with (2) brain structure information (cranial ultrasonography), and (3) perinatal and (4) postnatal risk factors for the prediction of death or neurodevelopmental impairment (NDI) in extremely preterm infants.

**Design, Setting, and Participants:**

Preterm newborns (23-28 weeks’ gestational age) admitted to the neonatal intensive care unit at Amiens-Picardie University Hospital were retrospectively included (January 1, 2013, to January 1, 2018). Risk factors from the 4 categories were collected during the first 2 weeks post delivery. Neurodevelopmental impairment was assessed at age 2 years with the Denver Developmental Screening Test II. No or moderate NDI was considered a favorable outcome. Death or severe NDI was considered an adverse outcome. Data analysis was performed from August 26, 2021, to March 31, 2022.

**Main Outcomes and Measures:**

After the selection of variables significantly associated with outcome, 4 unimodal prognostic models (considering each category of variable independently) and 1 multimodal model (considering all variables simultaneously) were developed. After a multivariate analysis for models built with several variables, decision-tree algorithms were run on each model. The areas under the curve for decision-tree classifications of adverse vs favorable outcomes were determined for each model, compared using bootstrap tests, and corrected for type I errors.

**Results:**

A total of 109 newborns (58 [53.2% male]) born at a mean (SD) gestational age of 26.3 (1.1) weeks were included. Among them, 52 (47.7%) had a favorable outcome at age 2 years. The multimodal model area under the curve (91.7%; 95% CI, 86.4%-97.0%) was significantly higher than those of the unimodal models (*P* < .003): perinatal model (80.6%; 95% CI, 72.5%-88.7%), postnatal model (81.0%; 95% CI, 72.6%-89.4%), brain structure model (cranial ultrasonography) (76.6%; 95% CI, 67.8%-85.3%), and brain function model (cEEG) (78.8%; 95% CI, 69.9%-87.7%).

**Conclusions and Relevance:**

In this prognostic study of preterm newborns, the inclusion of brain information in a multimodal model was associated with significant improvement in the outcome prediction, which may have resulted from the complementarity of the risk factors and reflected the complexity of the mechanisms that interfered with brain maturation and led to death or NDI.

## Key Points

**Question:**

Does a multimodal model combining perinatal, postnatal, and brain structure and function risk factors improve the prediction of death or neurodevelopmental impairment at age 2 years in extremely preterm infants?

**Findings:**

In a prognostic study including 109 extremely preterm newborns, multimodal processing of perinatal, postnatal, and brain structure and function risk factors significantly improved the prediction of 2-year outcome compared with unimodal models in which risk factors were processed independently.

**Meaning:**

This model for prediction of outcomes for extremely preterm newborns may be useful as a decision support tool in critical care management.

## Teaser

This prognostic study develops a multimodal model to predict the neurodevelopmental outcome of extreme preterm newborns at age 2 years.

## Introduction

Despite substantial improvements in neonatal care, the risk of death or neurodevelopmental impairment (NDI) in extremely preterm newborns remains high.^1,2^ The early assessment of a preterm newborn’s prognosis is of the utmost importance for making treatment decisions and providing parents with critically important information.^3^ In the longer term, the accurate identification of newborns with the highest risk of NDI would allow the implementation of early, personalized rehabilitation procedures to reduce neurocognitive deficiencies and handicaps.^4^

Many prognostic tools for quantifying the risk of death or NDI in preterm newborns have been developed.^3^ With few exceptions, the currently available models are limited by (1) their use of conventional analytical methods,^3^ (2) their reliance on a small number of clinical and laboratory variables, and (3) their failure to take account of prognostically valuable information^5,6,7^ on the brain’s structure^3,8,9,10,11,12^ and function.^13,14,15,16,17,18^ By using an innovative combination of unsupervised multivariate analyses and classification and regression tree analyses (CART) and by considering perinatal factors, postnatal factors, information on brain structure (cranial ultrasonography [cUS]), and brain function (conventional electroencephalography [cEEG]), we developed and evaluated a multimodal prognostic model for predicting the 2-year outcome in preterm newborns.

## Methods

### Patients

The preterm newborns meeting the following criteria were included: (1) born at 23 to 28 weeks’ gestational age (January 1, 2013, to January 1, 2018), (2) admitted to the neonatal intensive care unit (NICU) of Amiens-Picardie University Hospital, and (3) having undergone cEEG and cUS examination in the first 2 weeks after delivery. Newborns with genetic diseases or major malformations were excluded. Each parent gave written informed consent. The study was approved by the local investigational review board (CPP Nord-Ouest II). The study followed the Transparent Reporting of a Multivariable Prediction Model for Individual Prognosis or Diagnosis (TRIPOD) reporting guideline.

### Outcomes

For infants discharged from the NICU alive, pediatricians in the regional perinatal network assessed the neurodevelopmental outcome at age 2 years by administering the Denver Developmental Screening Test II (DDST II).^19^ Neurodevelopmental impairment was considered absent when more than 75% of the objectives were successfully achieved, moderate for a DDST II score between 50% and 75%, and severe for a DDST II score below 50%.

No or moderate NDI was considered a favorable outcome, severe NDI or death before discharge from the NICU was considered an adverse outcome. The circumstances of the death (limited use of life-sustaining therapies [LLST] or no LLST) were documented.

### Statistical Analysis

**Figure 1.**
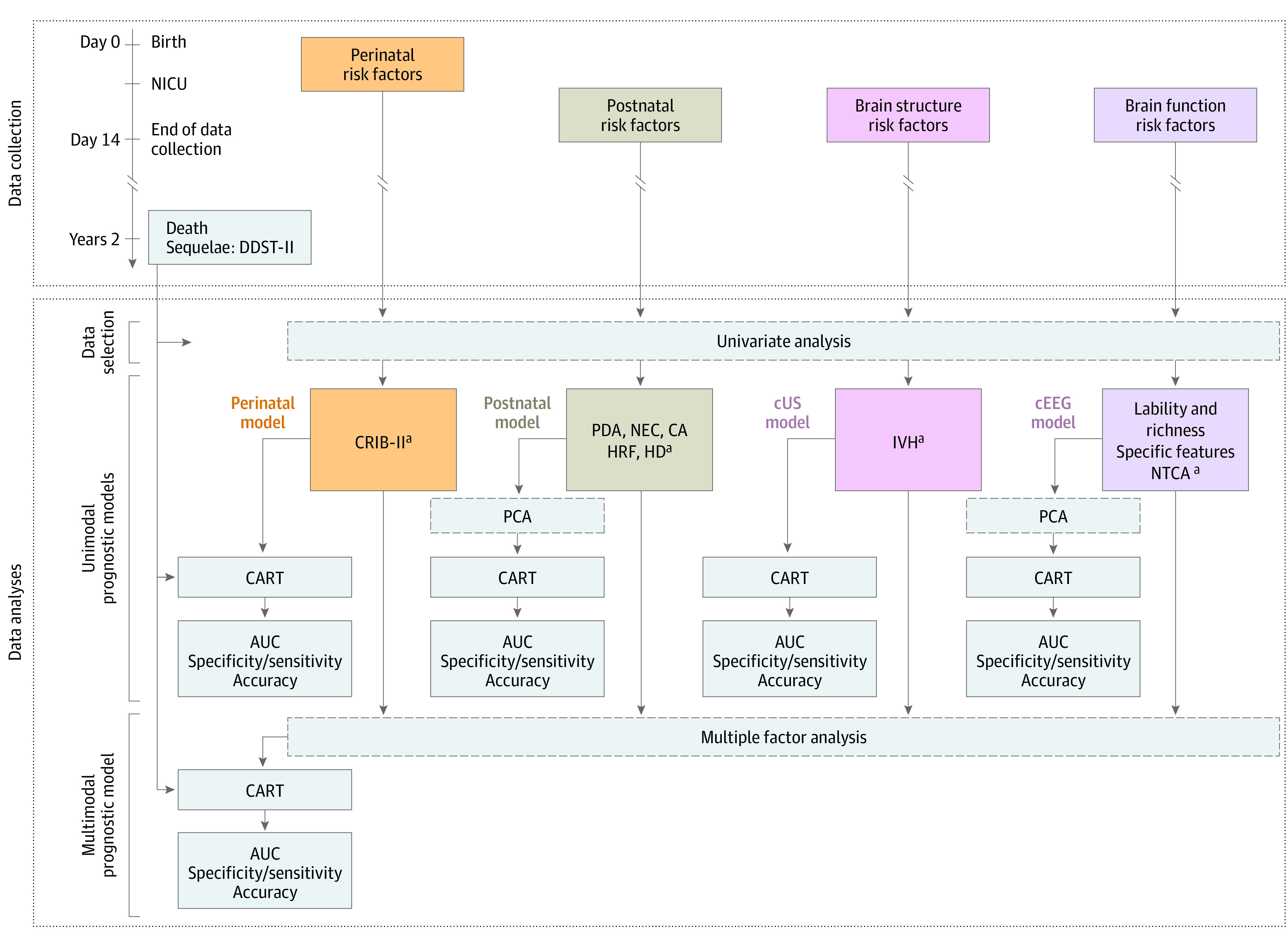
Study Design and Methods Classification and regression tree (CART), area under the curve (AUC), and prognostic performances were evaluated for favorable vs adverse outcome groups. CA indicates nonfatal cardiac arrest; cEEG, conventional electroencephalography; CRIB-II, Clinical Risk Index for Babies-II; cUS, cranial ultrasonography; DDST-II, Denver Developmental Screening Test II; HD, hemodynamic disorders; HRF, hypoxic respiratory failure; IVH, intraventricular hemorrhage; NTCA, negative theta central activity; NEC, necrotizing enterocolitis; NICU, neonatal intensive care unit; PCA, principal component analysis; and PDA, patent ductus arteriosus. ^a^Only variables selected in the univariate analyses were considered for each unimodal prognostic model.

The study was performed in 3 steps (Figure 1), and data analysis was conducted from August 26, 2021, to March 31, 2022. We selected variables from (1) perinatal, (2) postnatal, (3) brain structure (cUS), and (4) brain function (cEEG) risk factors collected during the first 2 weeks after delivery. Except for cEEG data, risk factors were collected from clinical reports; cEEG findings were interpreted by 2 experienced neurophysiologists (L.R., E.B.-P.) blinded to clinical and outcome information (except gestational age at EEG recording)^17,18,20^ (Table 1; eMethods 1 in Supplement 1). The risk factors associated with favorable vs adverse outcomes were selected in univariate analyses, using the Fisher exact test for binary variables and *t* test or Mann-Whitney test for ordinal and continuous variables. Depending on the statistical test, the data are reported as relative risk (RR) and its 95% CI, the mean and SD, or the median and IQR. Variables with a 2-sided, unpaired *P* value <.05 were discarded from model development.

**Table 1.** Risk Factor Scores and Univariate Statistical Analyses in the Favorable vs Adverse Outcome Groups

Risk factor	Scoring	Group		RR (95% CI)^a^	*P* value
		Favorable outcome (n = 52)	Adverse outcome (n = 57)		
Perinatal risk factors					
CRIB-II score, mean (SD)^21^^,^^b^	0 to 27	10.1 (2.4)	12.9 (2.2)	Difference: 2.8 (1.9 to 3.6)	<.001^c^
Gestational age, mean (SD)	wk	27.0 (1.4)	25.7 (1.0)	Difference: 1.3 (0.8 to 1.7)	<.001^c^
Birth weight, mean (SD)	g	886 (200)	730 (151)	Difference: 155 (89 to 222)	<.001^c^
Body temperature on admission to the NICU, median (IQR)	°C	36.6 (36.0 to 36.9)	35.8 (35.3 to 36.5)	Difference: 0.6 (0.3 to 1.0)	<.001^d^
Base excess on admission to the NICU, median (IQR)	mmoL/L	−5.2 (−2.6 to 6.5)	−6.9 (−4.5 to 9.4)	Difference: 1.8 (0.3 to 3.4)	.02^d^
Sex (male:female)^e^	ratio	24:28	34:23	1.3 (0.9 to 1.9)	.18^f^
Type of pregnancy (singleton = 0; multiple = 1), No.	0 to 1	Singleton: 8; multiple: 44	Singleton: 16; multiple: 41	2.0 (0.9 to 4.4)	.16^f^
Type of delivery (cesarean = 0; vaginal = 1), No.	0 to 1	Cesarean: 30; vaginal: 22	Cesarean: 30; vaginal: 27	1.1 (0.7 to 1.7)	.70^f^
Inborn/outborn status (inborn = 0; outborn = 1), No.	0 to 1	Inborn: 9; outborn: 43	Inborn: 16; outborn: 41	0.9 (0.7 to 1.1)	.25^f^
Antenatal corticosteroid therapy (no = 0; yes = 1), No.	0 to 1	No: 46; yes: 6	No: 42; yes: 15	2.3 (1.0 to 5.4)	.06^f^
Antenatal magnesium sulfate therapy (no = 0; yes = 1), No.	0 to 1	No: 11; yes: 41	No: 13; yes: 44	1.0 (0.8 to 1.2)	>.99^f^
Chorioamnionitis (no = 0; yes = 1), No.	0 to 1	No: 44; yes: 65	No: 44; yes: 13	1.5 (0.7 to 3.2)	.34^f^
Postnatal morbidities (0-14 d)					
Necrotizing enterocolitis (Bell classification), median (IQR)^22^	0 to 3	1 (0 to 2)	2 (0 to 2)	Difference: 1 (0 to 1)	.009^d^
		No.: 0 = 25; 1 = 7; 2 = 16; 3 = 4	No.: 0 = 16; 1 = 2; 2 = 34; 3 = 5		
Hypoxemic respiratory failure (no = 0; yes = 1), No.	0 to 1	No: 43; yes: 9	No: 22; yes: 35	3.5 (1.9 to 6.6)	<.001^f^
Hemodynamic disorders (no = 0; yes = 1), No.	0 to 1	No: 31; yes: 21	No: 10; yes: 47	2.0 (1.4 to 2.9)	<.001^f^
Nonfatal cardiac arrest (no = 0; yes = 1), No.	0 to 1	No: 51; yes: 1	No: 48; yes: 9	8.2 (1.1 to 62.6)	.02^f^
Hemodynamically significant patent ductus arteriosus (no = 0; yes = 1), No.	0 to 1	No: 26; yes: 26	No: 16; yes: 41	1.4 (1.1 to 2.0)	.03^f^
Blood infection with bacteremia (no = 0; yes = 1), No.	0 to 1	No: 24; yes: 28	No: 19; yes: 38	1.2 (0.9 to 1.7)	.24^f^
Structural brain injury (cUS) (0-14 d)					
Intraventricular hemorrhage (Papile classification) (none = 0; grade 1 = 1; grade 2 = 2; grade 3 = 3; grade 4 = 4), median (IQR)^23^	0 to 4	1 (0 to 2); No.: none: 25; grade 1: 9; grade 2: 8; grade 3: 7; grade 4, 3	3 (2 to 4); No.: none: 8; grade 1: 5; grade 2: 10; grade 3: 15; grade 4: 19	Difference: 2 (1 to 2)	<.001^d^
Periventricular leukomalacia (none = 0; periventricular echogenicity = 1; cystic lesion = 2), median (IQR)^24,25^	0 to 2	0 (0 to 1); No.: none: 38; periventricular echogenicity = 13; cystic lesion: 1	0 (0 to 0); No.: none: 45; periventricular echogenicity = 10; cystic lesion: 2	Difference: 0 (0 to 0)	.52^d^
Brain function risk factors (cEEG) (0-14 d)^16,17,18^					
Delta brush wave (normal = 0; insufficient occurrence = 1; disorganized = 2; absent = 3), median (IQR)	0 to 3	0 (0 to 1); No.: normal: 38; insufficient occurrence: 3; disorganized: 10; absent: 1	2 (0 to 2); No.: normal: 17; insufficient occurrence: 6; disorganized: 24; absent: 10	Difference: 2 (0 to 2)	<.001^d^
Theta occipital activity coalescing with a slow wave (normal = 0; disorganized = 1; absent = 2), median (IQR)	0 to 2	0 (0 to 1); No.: normal: 32; disorganized: 18; absent: 2	1 (0 to 1); No.: normal: 18; disorganized: 26; absent: 13	Difference: 1 (0 to 1)	.001^d^
Theta temporal activity coalescing with a slow wave (normal = 0; insufficient occurrence = 1; disorganized = 2; absent = 3), median (IQR)	0 to 3	1 (0 to 2); No.: normal: 23; insufficient occurrence: 6; disorganized: 23; absent: 0	2 (1 to 3); No.: normal: 12; insufficient occurrence:10; disorganized: 20; absent: 15	Difference: 1 (0 to 1)	.001^d^
Theta frontal activity coalescing with a slow wave (normal = 0; disorganized = 1; absent = 2), median (IQR)	0 to 2	1 (0 to 1); No.: normal: 21; disorganized: 29; absent: 2	1 (1 to 2); No.: normal: 14; disorganized: 28; absent: 15	Difference: 0 (0 to 1)	.005^d^
Lability (yes = 0; no = 1), No.	0 to 1	Yes: 51; no: 1	Yes: 41; no: 16	14.6 (2.0 to 106.2)	<.001^f^
Richness (normal = 0; abnormal = 1), No.	0 to 1	Normal: 49; abnormal: 3	Normal: 39; abnormal: 18	5.5 (1.7 to 17.5)	<.001^f^
Negative theta central activity (no = 0; yes = 1), No.	0 to 1	No: 45; yes: 7	No: 27; yes: 30	3.9 (1.9 to 8.1)	<.001^f^
Positive rolandic sharp waves (none = 0; ≤1/min = 1; >1/min = 2), median IQR)	0 to 2	0 (0 to 0); No.: none: 50; <1/min: 2; >1/min: 0	0 (0 to 0); No.: none: 52; <1/min: 3; >1/min: 2	Difference: 0 (0 to 0)	.29^d^
Electrographic seizures (no = 0; yes = 1), No.	0 to 1	No: 52; yes: 0	No: 53; yes: 4	NA	.12^f^
Temporal sharp waves (no = 0; yes = 1), No.	0 to 1	No: 51; yes: 1	No: 50; yes: 7	6.4 (0.8 to 50.2)	.06^f^
Occipital sharp waves (no = 0; yes = 1), No.	0 to 1	No: 51; yes: 1	No: 53; yes: 4	3.6 (0.4 to 31.6)	.37^f^
Frontal sharp waves (no = 0; yes = 1), No.	0 to 1	No: 41; yes: 11	No: 43; yes: 14	1.2 (0.6 to 2.3)	.82^f^

Abbreviations: CRIB-II, Clinical Risk Index for Babies II; cEEG, conventional electroencephalogram; cUS, cranial ultrasonography; NA, not applicable; NICU, neonatal intensive care unit; RR, relative risk.

^a^ RR = (multiple / [multiple + singleton] adverse group) / (multiple / [multiple + singleton] favorable group) for pregnancy type.

^b^ The CRIB-II score is calculated from the following indicators: the gestational age at birth, birth weight, sex, base excess, and body temperature on admission to the NICU. No missing data.

^c^ *t* test.

^d^ Fisher exact test.

^e^ Sex ratio: (male / [male + female] adverse group) / (male / [male + female] favorable group).

^f^ Mann-Whitney test.

#### Unimodal Prognostic Models

We built unimodal prognostic models for each category of selected risk factors and determined their respective prognostic performances in a CART analysis^26^ (eMethods 2 in Supplement 1). CART algorithms were directly applied to categories with a single risk factor selected. For categories comprising several risk factors, a CART algorithm was applied to the first 3 principal axes (PAs) of a principal component analysis (PCA)^27^ previously performed. Principal component analysis decomposes the global variance of the original variable set into new linear orthogonal variables (ie, PAs). Each of the original variables explains a various part (ie, contribution) of this global variance. The contribution of each original variable is defined transparently by a ratio (percentage) and a vector (direction) (eMethods 3 in Supplement 1). The area under the curve (AUC), specificity, sensitivity, positive predictive values (PPVs), negative predictive values (NPVs), and accuracy were calculated for the CART partitions for outcome in each model.

#### Multimodal Prognostic Model

We built a multimodal prognostic model by simultaneously considering all the selected risk factors. The prognostic performance of the multimodal model was estimated as described above, except that the CART algorithm was applied to the PAs from a multiple factor analysis (MFA)^28,29^ (eMethods 4 in Supplement 1). The MFA is an extension of the PCA in which the initial set of variables is structured into groups (perinatal, postnatal, cUS, and cEEG) and weighted to mitigate the influence of differences in the number of variables per group. The contribution of each group of variables is determined as for PCA. A complementary MFA tested the potential bias of LLST circumstances.

We compared the respective performance levels of the unimodal and multimodal models. Pairwise comparisons of AUCs from the unimodal and multimodal models were performed with bootstrap tests and corrected for type I errors for multiple comparisons with the false discovery rate (*P* < .05; *k* = 10).

#### Reproducibility and Cross-Validations of the Multimodal Model

A support vector machine classification of the outcomes was added to the CART classification for the MFA (eMethods 5 in Supplement 1). The overfitting of CART and support vector machine classification accuracies was tested with 10-fold cross-validations and completed by a split cross-validation (74 and 35 newborns split into training and validation subsets) (eMethods 6 in Supplement 1). Statistical analyses were performed with R, version 4.0.4 software (R Foundation for Statistical Computing).^30^

## Results

### Population

Among the 318 preterm newborns born at 23 to 28 weeks’ gestational age admitted in the NICU, 109 met the inclusion criteria (eResults 1 in Supplement 1). The mean (SD) gestational age at delivery was 26.3 (1.1) weeks, 58 (53.2%) were male, 51 (46.8%) were female, and the male to female ratio was 1.4. Fifty (45.9%) of the 109 newborns died in the NICU. Twenty-six (52.0%) of the 50 deaths followed LLST. No death was reported between the NICU discharge and age 2 years. Of the 59 (54.1%) surviving infants, 39 (66.1%) did not show any NDI, 13 (22.0%) showed moderate NDI, and 7 (11.9%) had severe NDI. Overall, 52 of the 109 newborns (47.7%) had a favorable outcome, and 57 (52.3%) had an adverse outcome (Table 1).

The mean (SD) of Clinical Risk Index for Babies-II (CRIB-II) score was 11.7 (2.3). The most frequent postnatal morbidities were hemodynamic disorders (68 [62.4%]), hemodynamically significant patent ductus arteriosus (67 [61.5%]), and blood infection with bacteremia (66 [60.6%]). Other morbidities in the 109 newborns comprised necrotizing enterocolitis (59 [54.1%]), hypoxic respiratory failure (44 [40.4%]), and nonfatal cardiac arrest (10 [9.2%]). Forty-four (40.4%) newborns had high-grade intraventricular hemorrhage (grade ≥3) and 84 (77.1%) had cEEG abnormalities: 80 (73.3%) had background abnormalities and 51 (46.8%) had superimposed pathologic features (median age at cEEG: 4 days [IQR, 3-7 days]). Of these 51 newborns, 37 (72.5%) had negative theta central activity, and 7 (13.7%) had positive rolandic sharp waves (PRSWs) (Table 1).

### Unimodal Model Prognostic Performances

The unimodal models tested the 4 risk factor categories independently of each other (Table 1 and Table 2). Complementary statistics are presented in eResults 2 in Supplement 1.

**Table 2.** The Performance Levels of Unimodal and Multimodal Prognostic Models in the Adverse and Favorable Outcome Groups

Risk factor	% (95% CI)						*P* value for pairwise comparisons of models^a^			
	Accuracy	Specificity	Sensitivity	PPV	NPV	AUC	Perinatal (CRIB-II)	Postnatal (PCA1)	Brain structure (IVH)	Brain function cEEG (PCA2)
Unimodal models										
Perinatal model (CRIB-II)	71.6 (62.1-79.8)	92.0 (74.0-99.0)	65.5 (54.3-75.5)	96.5 (87.9-99.6)	44.2 (30.5-58.7)	80.6 (72.5-88.7)	NA	NA	NA	NA
Postnatal model (PCA1)	79.8 (71.1-86.9)	77.8 (64.4-88.0)	81.8 (69.1-90.9)	78.9 (66.1-88.6)	80.8 (67.5-90.4)	81.0 (72.6-89.4)	.94	NA	NA	NA
Brain structure model (IVH)	71.6 (62.1-79.8)	72.3 (64.4-88.0)	71.0 (58.1-81.8)	77.2 (64.2-87.3)	65.4 (50.9-78.0)	76.6 (67.8-85.3)	.48	.44	NA	NA
Brain function model (cEEG) (PCA2)	77.1 (68.0-84.6)	74.5 (61.0-85.3)	79.6 (66.5-89.4)	75.4 (62.2-85.9)	78.8 (65.3-88.9)	78.8 (69.9-87.7)	.75	.71	.70	NA
Multimodal model										
All risk factors (MFA)	87.2 (79.4-92.8)	88.0 (75.6-95.5)	86.4 (75.0-94.0)	89.5 (78.5-96.0)	85.5 (71.9-93.1)	91.7 (86.4-97.0)	.002	<.001	<.001	.003

Abbreviations: AUC, area under the curve; cEEG, conventional electroencephalogram; CRIB-II, Clinical Risk Index for Babies II; IVH, intraventricular hemorrhage; MFA, multiple factor analysis; NA, not applicable; NPV, negative predictive value; PCA, principal component analysis; PPV, positive predictive value.

^a^ Bootstrap test; all *P* values were statistically significant after false discovery rate correction (*k* = 10; *P* < .05).

#### Perinatal Model

The single risk factor included in the perinatal model was the CRIB-II score, based on gestational age at birth, birth weight, sex, base excess, and body temperature on NICU admission.^21^ The CART classification for outcome produced the following findings: AUC, 80.6% (95% CI, 72.5%-88.7%); specificity, 92.0% (95% CI, 74.0%-99.0%); sensitivity, 65.5% (95% CI, 54.3%-75.5%); PPV, 96.5% (95% CI, 87.9%-99.6%); NPV, 44.2% (95% CI, 30.5%-58.7%); and accuracy, 71.6% (95% CI, 62.1%-79.8%). A total of 78 newborns were correctly classified and 31 were misclassified.

#### Postnatal Model

The risk factors included in the postnatal model were hypoxic respiratory failure, patent ductus arteriosus, nonfatal cardiac arrest, hemodynamic disorders, and necrotizing enterocolitis. The CART classification for outcome for the first 3 PAs previously calculated by PCA (PCA1) gave the following findings: AUC, 81.0% (95% CI, 72.6%-89.4%); specificity, 77.8% (95% CI, 64.4%-88.0%); sensitivity, 81.8% (95% CI, 69.1%-90.9%); PPV, 78.9% (95% CI, 66.1%-88.6%); NPV, 80.8% (95% CI, 67.5%-90.4%); and accuracy, 79.8% (95% CI, 71.1%-86.9%). A total of 87 newborns were correctly classified and 22 were misclassified.

#### Brain Structure Model

The single risk factor included in the brain structure model (cUS) was the intraventricular hemorrhage grade. The CART classification for outcome gave the following findings: AUC, 76.6% (95% CI, 67.8%-85.3%); specificity, 72.3% (95% CI, 64.4%-88.0%); sensitivity, 71.0% (95% CI, 58.1%-81.8%); PPV, 77.2% (95% CI, 64.2%-87.3%); NPV, 65.4% (95% CI, 50.9%-78.0%); and accuracy, 71.6% (95% CI, 62.1%-79.8%). A total of 78 newborns were correctly classified and 31 were misclassified.

#### Brain Function Model

The risk factors included in the brain function model (cEEG) were the richness and lability, the characteristics of age-related features, and the negative theta central activity. The CART classification for outcome on the first 3 PAs previously calculated by PCA (PCA2) gave the following findings: AUC, 78.8% (95% CI, 69.9%-87.7%); specificity, 74.5% (95% CI, 61.0%-85.3%); sensitivity, 79.6% (95% CI, 66.5%-89.4%); PPV, 75.4% (95% CI, 62.2%-85.9%); NPV, 78.8% (95% CI, 65.3%-88.9%); and accuracy, 77.1% (95% CI, 68.0%-84.6%). A total of 84 newborns were correctly classified and 25 were misclassified.

### Prognostic Performance of the Multimodal Model

**Figure 2.**
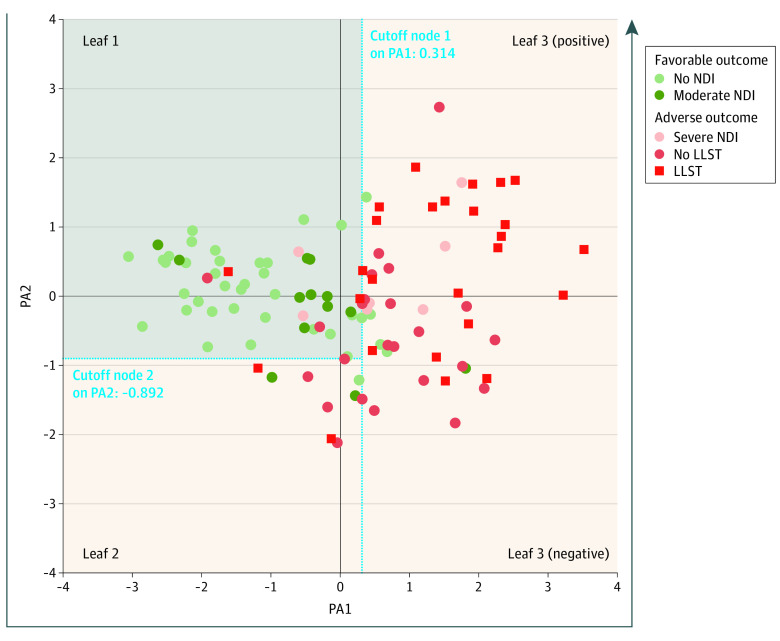
Newborn Distribution According to Principal Axis (PA) 1 and PA2 Multiple Factor Analysis (MFA) and Classification and Regression Tree (CART) for Outcome Partition A total of 109 newborns included according to the outcome. The position of each newborn was defined by individual coordinates for PA1 and PA2. Leaf 1, leaf 2, and leaf 3 were categorized using the CART partition determined by the 2 cutoff nodes at PA1 and PA2. Newborns were unequally distributed according to the outcome, reflecting a gradient of severity from leaf 1 to leaf 3 (positive): leaf 1, favorable outcome newborns mainly on the left (lowest PA1 values) and adverse outcome newborns on the right (highest PA1 values: leaf 2, limited use of life-sustaining therapies (LLST) newborns mainly on the upper right (highest PA1 and positive PA2 values: leaf 3, [positive]) and no LLST newborns on the lower right (highest PA1 and negative PA2 values) (leaf 3 [negative]). The arrow from the top of the left y-axis down to the x-axis and up the right y-axis indicates the gradient of severity from leaf 1 to leaf 3. NDI indicates neurodevelopmental impairment.

**Figure 3.**
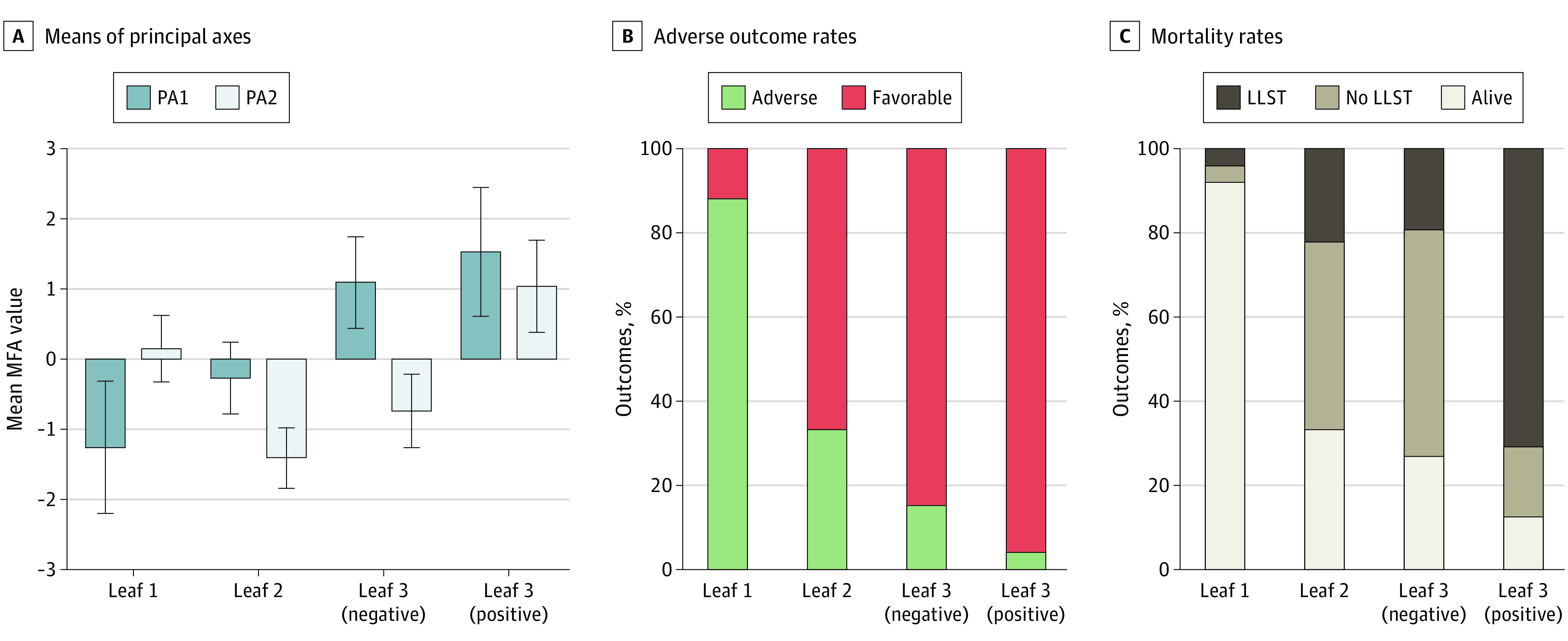
Classification and Regression Tree Partition on Multiple Factor Analysis (MFA) and Outcome Proportions Principal axis (PA) PA1 and PA2 means and SD (A), favorable vs adverse outcome proportions (B), and death (limited use of life-sustaining therapies [LLST] vs no LLST) vs alive at discharge proportions (C) for newborns classified in leaf 1, leaf 2, leaf 3 (negative), and leaf 3 (positive). PA1 means increase, reflecting the accumulation of the risk factors from leaf 1 to leaf 3 (positive) (A), was associated with an increase of adverse outcome (B) and mortality (C) rates. Negative PA2 means (A) (leaf 2 and leaf 3 [negative]), reflecting worse perinatal and postnatal morbidities indicators, were associated with a higher risk of no LLST death (C). Positive PA2 means (A) leaf 3 (positive), reflecting worse brain structure and function indicators, was associated with a higher risk of LLST death.

The multimodal model simultaneously combined the 4 risk factor categories in an MFA (Table 1 and Table 2, Figure 2 and Figure 3; eResults 2 in Supplement 1).

#### MFA and Variable Contribution

The 4 groups of risk factors—perinatal (CRIB-II), postnatal (hypoxic respiratory failure, patent ductus arteriosus, nonfatal cardiac arrest, hemodynamic disorders, and necrotizing enterocolitis), brain structure (intraventricular hemorrhage), and brain function (richness and lability, characteristics of age-related specific features, and negative theta central activity)—contributed in similar proportions and direction to the first PA of the MFA (PA1 MFA) (perinatal, 28.2%; postnatal, 24.1%; brain structure, 25.5%; and brain function, 22.2%) (eResults 3 in Supplement 1). Thus, the PA1 MFA resulted from the accumulation and aggregation of all groups of risk factors (the worse the risk factors, the higher the PA1 MFA values).

The groups of risk factors contributed in 2 directions to the second PA (PA2 MFA). The main contributions to the negative pole of PA2 MFA were provided by postnatal (36.7%) and perinatal (11.7%) risk factors (the worse the postnatal and perinatal indicators, the lower the PA2 MFA values). The main contributions to the positive pole of PA2 MFA were provided by the brain function (41.3%) and structure (10.3%) risk factors (the worse the cEEG and intraventricular hemorrhage indicators, the higher the PA2 MFA values).

### CART Partition for Outcome

The CART for outcome analysis applied to the MFA identified 3 leaves from 2 nodes (Figure 2). Leaf 1 included 50 (45.9%) of the 109 newborns who scored below 0.314 on PA1 MFA (cutoff node 1) and above −0.892 on PA2 MFA (cutoff node 2). Leaf 2 included 9 newborns (8.3%) who scored below 0.314 on PA1 MFA and below −0.892 on PA2 MFA. Leaf 3 included 50 newborns (45.9%) with a score above 0.314 on PA1 MFA. Among the leaf 3 group, 26 had negative values for PA2 MFA (negative pole of leaf 3) and 24 had positive values for PA2 MFA (positive pole of leaf 3).

The probability of an adverse outcome increased continuously from leaf 1 to leaf 3 (positive) (adverse outcomes: leaf 1, 12%; leaf 2, 67%; leaf 3 [negative], 85%; and leaf 3 [positive], 96%; mortality: leaf 1, 8%; leaf 2, 67%; leaf 3 [negative], 73%; and leaf 3 [positive], 87%). This increase reflected the accumulation of the risk factors as evidenced by the continuous increase of PA1 MFA mean (SD) values: leaf 1, −1.3 (0.9); leaf 2, −0.3 (0.5); leaf 3 (negative), 1.1 (0.7); and leaf 3 (positive), 1.5 (0.9) (Figure 3).

Circumstances of death (LLST vs no LLST) were mainly associated with the polarity of PA2 MFA. Positive PA2 values (PA2 mean [SD], 1.0 [0.7]) were associated with a higher proportion of LLST (71%) vs no LLST (17%) death (leaf 3 [positive]) and were related to worse cEEG and intraventricular hemorrhage indicators. Negative PA2 values (PA2 mean [SD], −0.7 [0.5]) were associated with a higher proportion of no LLST (54%) vs LLST (19%) death and associated with worse perinatal and postnatal morbidity indicators (leaf 3 [negative]). Similarly, the lower proportion of LLST (22%) vs no LLST (42%) death in leaf 2 was associated with negative values of PA2 MFA (PA2 mean [SD],  −1.4 [0.4]). Newborns who died before discharge with LLST (4%) or no LLST (4%) were marginal in leaf 1 (PA2 mean [SD], 0.01 [0.5]).

### Multimodal Prognostic Performances

The CART classification for outcome for the first 3 PA MFA values gave a very high AUC, 91.7% (95% CI, 86.4%-97.0%); specificity, 88.0% (95% CI, 75.6%-95.5%); sensitivity, 86.4% (95% CI, 75.0%-94.0%); PPV, 89.5% (95% CI, 78.5%-96.0%); NPV, 85.5% (95% CI, 71.9%-93.1%); and accuracy, 87.2% (95% CI, 79.4%-92.8%). A total of 95 newborns were correctly classified and 14 were misclassified. The complementary MFA performed after exclusion of LLST newborns gave a quite similar high AUC, 88.7% (95% CI, 81.4%-96.1%) to the MFA in the entire population (eResults 4 in Supplement 1). The AUC of the multimodal model (91.7%) was significantly higher than that of unimodal models: the perinatal model (80.6%; *P* = .002), postnatal model (81.0%; *P* < .001), brain structure model (76.6%; *P* < .001), and brain function model (78.8%; *P* < .003).

### Reproducibility and Cross-Validations of the Multimodal Model

The support vector machine classification of outcomes gave an accuracy of 89.0%. The 10-fold cross-validations of accuracy for the CART classification in the multimodal model (85.0%) and for the support vector machine classification (88.5%) were satisfactory (eResults 5 in Supplement 1). The AUCs for the split cross-validation were quite similar for the training subset (91.1%; 95% CI, 87.5%-100%) and validation subset (94.3%; 95% CI, 73.6%-96.3%) (eResults 6 in Supplement 1).

## Discussion

Predicting the neurodevelopmental outcome of extremely preterm infants is a daily challenge for physicians and a prominent concern for parents.^3,13^ Given the complexity of prognostic factors assessed in the clinic setting, the prognosis varies from one physician to another^31^; hence, a reliable prognostic model would be a highly valuable clinical tool.^3^ Although numerous prognostic models have been developed, there are still important prospects for improvement of risk factors evaluated.^3,11,32,33^ With few exceptions, existing models were limited by the small number of variables and the use of conventional analytic methods ill-equipped to handle a complex data set.^3^ Our study addressed the need for new models analyzing a large and original data set of risk factors, including variables from brain function not necessarily linearly associated with the outcome.^3^ We considered recommendations by Linsell and collaborators^34^ and other researchers^11^ and were especially careful to avoid black box models (eg, neural networks) unable to show the respective contributions of risk factors, which would be an obstacle for use in routine clinical practice.

The prognostic performances of the unimodal models in this study were similar to each other and similar to those reported in the literature.^3^ The composite CRIB-II score reflecting adaptation to extrauterine life is frequently used to predict death.^21^ Given its fair sensitivity, this tool cannot alone predict the neurodevelopmental outcome with sufficient accuracy.

Postnatal morbidities gave satisfactory sensitivity and specificity values for predicting the outcome, as did the intraventricular hemorrhage model. Premature birth induces a sudden rupture from the uterine environment in a critical period of development. The immature organs are exposed to various harmful extrauterine factors, increasing their risk of failure, which in turn increases the likelihood of structural and functional brain disorders and NDI.^35,36^

The present study noted that general organization (richness and lability), the negative theta central activity (hitherto of undetermined pathologic significance), and age-related features contributed equally to the good prognostic performance of cEEG, which remains the highest standard for neonatal brain function assessment.^7,16,17,18,37,38,39,40,41,42,43^ Surprisingly, PRSWs (cEEG) and related cystic periventricular leukomalacia (cUS) were not significantly associated with the outcome.^41,44,45,46,47,48,49^ However, the low prevalence of PRSWs and cystic periventricular leukomalacia in this study population (which may be in part explained by the difficulty in diagnosing periventricular leukomalacia before 30 weeks’ gestational age^41,50^ and by improvements in perinatal care^6,7^) might have contributed to the lack of statistical significance.

Combining all the risk factors greatly increased the prediction accuracy. Our comprehensive multimodal model assessed all the complex mechanisms that interfere with brain maturation and lead to NDI.^51^ Good adaptation to extrauterine life, absence of severe postnatal morbidities and high-grade intraventricular hemorrhage, and presence of normal or moderately altered cEEG activity within the first 2 weeks after delivery made equal contributions to a highly accurate prediction of survival and the absence of severe NDI. Poor status for 1 or more of these markers led to a worse prognosis in 2 ways. First, the combination of severe perinatal factors with major organ failure resulted in a high probability of an adverse outcome, including death despite resuscitation efforts. For these newborns, information on the brain status did not contribute markedly to the prognosis. Second, the combination of poor brain structure and function markers was also highly predictive of death—predominantly in the context of LLST—and severe NDI for survivors. These newborns did not have severe perinatal and postnatal markers, and postnatal markers did not contribute greatly to the prognosis.^52^ Our results emphasize the added value of functional brain information in a multimodal approach for the prediction of both favorable and adverse outcomes, especially in newborns with low-severity perinatal and postnatal indicators.

### Limitations

This study has limitations. First, despite cross-validations, the results of our single-center study in a population with a high mortality rate^1^ will have to be validated and generalized in an independent, multicenter population prior to clinical application. Second, except for the CRIB-II score, none of the perinatal risk factors were significantly associated with the outcome. These results might confirm their low prognostic value but might also be due to their low frequency and variability in our population and thus a lack of statistical power.^32,53,54^ Similar considerations apply to cystic periventricular leukomalacia and pathologic cEEG features (PRSWs) classically associated with a poor prognosis.

The high death rate raises the question of whether the LLST decision competes with and biases the prevalence of subsequent NDI. However, excluding infants who died after the LLST decision, the performances of the multimodal model were not obviously affected.

Despite these limitations, our results support previous recommendations to include cEEG data in prognostic models.^13,14^ The use of predictive models that include functional brain information might be restricted by limited access to expert cEEG technicians and neurophysiologists; this might justify the creation of a local or regional network,^55^ the development of a standardized procedure for cEEG interpretation, and the use of automated tools for greater reproducibility.^18,56,57^

Finally, the dynamic nature of maturation and subsequent complications should be considered. The life course framework model describes how the accumulation of risk at critical periods can impact subsequent health outcomes.^58^ The incorporation of sequential physiologic markers might enable the definition of individual prognostic outcome trajectories, which should be more valuable than a prediction made at a single, initial time point.^32^ This dynamic, predictive approach might help to identify newborns with the highest risk of developing subtle functional neurocognitive impairment diagnosed later in a neuropsychological assessment.^59^

## Conclusions

In this prognostic study of extremely preterm newborns, we used innovative statistical tools to develop a multimodal prognostic model from a complex set of very early risk factors that provided excellent performances. The gain in accuracy compared with unimodal models resulted from the complementary nature of the risk factors and reflected the complexity of the mechanisms that interfered with brain maturation and led to death or NDI. The validation of our model in a multicenter, prospective study could form the basis for the development of a clinically relevant, collective, decision support tool in critical care management.

## Notes

Supplement 1. **eMethods 1.** Risk-Factor Definition and Analytical Modalities **eMethods 2.** Classification and Regression Trees (CART) **eMethods 3.** Principal Component Analysis (PCA) **eMethods 4.** Multiple Factor Analysis (MFA) **eMethods 5.** Support Vector Machine (SVM) Classification **eMethods 6.** 10K-Fold Cross-Validation and Split Subsets Cross-Validation **eResults 1.** Demography of the Sample - Amiens and French Populations Comparison **eResults 2.** Additional Statistics: Comparisons of Favorable/Adverse Outcome and LLST/No.LLST Groups for Variables of Unimodal and Multimodal Models **eResults 3.** Additional Information on Variable Contributions to the MFA **eResults 4.** Additional MFA Excluding the Newborns Died in LLST Circumstances **eResults 5.** Additional Statistics: SVM and 10k-Fold Internal Cross-Validation **eResults 6.** Additional Statistics: Split Subsets Cross-Validation
eReferences

Supplement 2.
Data Sharing Statement
