# Supplementary material for: Predicting the Neurodevelopmental Outcome in Extremely Preterm Newborns Using a Multimodal Prognostic Model Including Brain Function Information
Source: JAMA Netw Open. 2023 Mar 8;6(3):e231590. doi: 10.1001/jamanetworkopen.2023.1590 (PMC9996404; doi:10.1001/jamanetworkopen.2023.1590)
Supplement: Supplement 1. — eMethods 1. Risk-Factor Definition and Analytical Modalities eMethods 2. Classification and Regression Trees (CART) eMethods 3. Principal Component Analysis (PCA) eMethods 4. Multiple Factor Analysis (MFA) eMethods 5. Support Vector Machine (SVM) Classification eMethods 6. 10K-Fold Cross-Validation and Split Subsets Cross-Validation eResults 1. Demography of the Sample - Amiens and French Populations Comparison eResults 2. Additional Statistics: Comparisons of Favorable/Adverse Outcome and LLST/No.LLST Groups for Variables of Unimodal and Multimodal Models eResults 3. Additional Information on Variable Contributions to the MFA eResults 4. Additional MFA Excluding the Newborns Died in LLST Circumstances eResults 5. Additional Statistics: SVM and 10k-Fold Internal Cross-Validation eResults 6. Additional Statistics: Split Subsets Cross-Validation eReferences [file jamanetwopen-e231590-s001.pdf]

Supplementary Online Content

Routier L, Querne L, Ghostine-Ramadan G, et al. Predicting the neurodevelopmental outcome in extremely preterm newborns using a multimodal prognostic model including brain function information. *JAMA Netw Open*. 2023;6(3):e231590. doi:10.1001/jamanetworkopen.2023.1590

eMethods 1. Risk-Factor Definition and Analytical Modalities

eMethods 2. Classification and Regression Trees (CART)

eMethods 3. Principal Component Analysis (PCA)

eMethods 4. Multiple Factor Analysis (MFA)

eMethods 5. Support Vector Machine (SVM) Classification

eMethods 6. 10K-Fold Cross-Validation and Split Subsets Cross-Validation

eResults 1. Demography of the Sample **-** Amiens and French Populations Comparison

eResults 2. Additional Statistics: Comparisons of Favorable/Adverse Outcome and LLST/No.LLST Groups for Variables of Unimodal and Multimodal Models

eResults 3. Additional Information on Variable Contributions to the MFA

eResults 4. Additional MFA Excluding the Newborns Died in LLST Circumstances

eResults 5. Additional Statistics: SVM and 10k-Fold Internal Cross-Validation

eResults 6. Additional Statistics: Split Subsets Cross-Validation

eReferences

This supplementary material has been provided by the authors to give readers additional information about their work.

eMethods-1. Risk-factor definition and analytical modalities

Perinatal variables

The Clinical Risk Index for Babies II (CRIB-II) is a score widely used by physicians to predict the initial risk of mortality on the basis of the following clinical and laboratory characteristics: gestational age, sex, birth weight, body temperature, and base excess value on admission to the NICU[1].

Chorioamnionitis was defined as the presence of fever (body temperature > 38°C) and fulfillment of at least two of the following criteria: elevated maternal C-reactive protein, leukocytosis, malodorous fluid and maternal or fetal tachycardia, and uterine tenderness[2,3].

Postnatal morbidities (in the 14 days after delivery)

- Bell's classification was used to assess necrotizing enterocolitis (NEC). Grade ≥ 2 NEC is considered to be a severe postnatal morbidity[4].
- Hypoxic respiratory failure (HRF) was defined as the presence of at least one of the following characteristics: FiO2 ≥ 60% or positive expiratory pressure (PEP) ≥ 6 cmH2O for 2 hours, the use of high frequency oscillations ventilation or inhaled nitric oxide.
- Hemodynamic disorders (HDs) which required the use of vaso-active drugs were considered.
- Non-fatal cardiac arrest (CA) was defined as a lasting decrease of the heart rate under 60/minute not resulting in death[5].

Brain structure risk-factors (according to cranial ultrasound, cUS) (in the 14 days after delivery)

- The presence/absence and severity of intraventricular hemorrhage (IVH) were scored using Papile's classification[6].
  0 = no IVH.
  1 = IVH limited to the germinal matrix.
  2 = IVH that fills less than 50% of the ventricles.
  3 = IVH with extension into the dilated ventricle.
  4 = IVH with parenchymal extension.
- Periventricular leukomalacia (PVL) was graduated with the following PVL score, adapted from the literature[7,8]:
  0 = no PVL.
  1 = periventricular echogenicity.
  2 = one or more cystic periventricular lesions.

Functional brain risk-factors (Conventional EEG (cEEG)) (in the 14 days after delivery)

All conventional EEGs were interpreted and classified by two experienced neurophysiologists (LR and EPB). They were both blinded to clinical information –except gestational age at birth and at EEG recording– and blinded to the outcome. The cEEG signal was analyzed by considering the general organization of electrical neural activities, the characteristics of specific maturational EEG features, and superimposed pathological features and seizures.

General organization of the EEG

- Lability was defined as the presence of fluctuations during quiescent (discontinuous) periods and active periods (bursts of activity) and amplitude/frequency modulations of the EEG features[9–11]. Lability was scored as normal (0) or absent (1).
- Richness was assessed in terms of the proportion and duration of quiescent periods (QPs) (interburst intervals) and bursts of activity, as a function of the postmenstrual age (PMA). Richness was considered to be normal if the QPs lasted less than 35 s and the bursts of activity lasted for more than 10 s for cEEG recordings acquired at 24-28 weeks of PMA (wPMA). For cEEG recordings acquired at 29-30 wPMA, richness was considered to be normal if the QPs lasted less than 20s and the bursts of activity lasted for more than 20s[9]. Richness was scored as normal (0) or insufficient (1).

Specific maturational features in EEG

The localization and morphology of the maturational EEG features (theta occipital, temporal, and frontal activities coalescing with a slow wave (TOA-SW, TTA-SW, and TFA-SW, respectively), and delta waves) were analyzed. TTA-SW, TFA-SW and TOA-SW were characterized by bursts of sharp 4-7 Hz waves coalescing with a biphasic slow wave, with an amplitude of 100-400 µV, localized in the temporal, frontal, and occipital regions, respectively[9–11]. Normally, TTA-

© 2023 Routier L et al. *JAMA Network Open.*

SW appears with a frequency of at least 1 per 2 min at 24-25 wPMA and 1 per min at 26-30 wPMA.. Delta waves were defined as monophasic or biphasic slow waves (0.5-1 Hz) that were smooth or superimposed with fast rhythms (5-25 Hz), with an amplitude of 100-400 µV and which appeared in sequences of several seconds[9–12]. Disorganized theta activities coalescing with a slow wave were defined as a deformed pattern with at least one of the following criteria: amplitude ≥ 500µV, fast activities < 4Hz or >7 Hz, sharp theta activities, and the absence of slow wave[13–15]. Delta waves were considered to be disorganized if they were deformed and met at least one of the following criteria: lack of smoothness, a wider base, frequency < 0.5 Hz, amplitude > 500 µV, a mechanical cogwheel appearance, or with fast activities invading the whole slow wave[13–15]. An insufficient occurrence of TTA-SW was defined as < 2/min at 24-26 wPMA and < 1/min at 27-30 wPMA. Delta brushes were insufficient if they appeared singly or at a frequency below 1/min.

- For TOA-SW and TFA-SW, the scores ranged from 0 to 2 (normal=0; disorganized=1; absent=2).
- For TTA-SW and Delta wave, the scores ranged from 0 to 3 (normal=0; insufficient occurrence=1; disorganized=2; absent=3).

Pathological features and seizures

- Positive rolandic sharp waves (PRSW) were defined as sharp waves with a positive polarity in the central areas (amplitude > 25 µV and duration < 500 ms). They were scored as absent (0), present with a density < 1/min (1), or present with a density > 1/min (2)[13,16–18].
- Temporal sharp waves, occipital sharp waves, and frontal sharp waves were defined as sharp waves in temporal, occipital and frontal regions, respectively, with an amplitude > 25 µV and duration < 500 ms. Each sharp waves were scored as absent (0) or present (1)[13].
- Negative theta central activity (NTCA)[13] was defined as isolated or sequential theta activity with a negative polarity in central regions (EEG electrodes: C4-C3-Cz) and an amplitude greater than 25 µV. NTCA were scored as absent (0) or present (1).
- Electrographic seizures were defined as repetitive rhythmic activity lasting for more than 10s and were scored as absent (0) or present (1)[19].

© 2023 Routier L et al. *JAMA Network Open.*

eMethods-2. Classification and regression trees (CART)

The CART[5-7] is a recursive algorithm that determines optimal cut-off points in variables –either unitary variables or principal axes resulting from multivariate analyses (principal component analyses and a multiple factor analysis in the present study)– that best separate cases from non-cases (adverse from favorable outcomes)[20–22]. The first split (node-1) is given by the cut-off that obtains the lower Gini index from among all variables entered in the model. This index varies between 0 and 1, with 0 meaning that all cases belong to one class and all non-cases belong to the other class. The procedure is repeated on the two outcome groups resulting from the first split and then for each new split. Only the branches of the tree's nodes that improve the correct classification survive the test. The terminal branches are called "leafs".

CART algorithm was run using the "Partykit" and "rpart" packages in R[23,24].

© 2023 Routier L et al. *JAMA Network Open.*

eMethods-3. Principal component analysis (PCA)

The PCA is a mathematical tool that summarizes the initial variables into a limited number of new, composite, linear variables (the principal axes (PAs)), which accurately reflect commonalities/discrepancies between newborns with regard to the initial variables[25]. The PCA decomposes the variance of the set of variables into orthogonal PAs, to which all the initial variables contribute to varying degrees. PAs are calculated in a similar way to a linear correlation (X/Y) but as applied to k variables. The first PA (PA1) is the linear solution that maximizes the sum of the individual squared distances (with initial variables) from the origin. Thus, PA1 carries the largest possible variance (eigenvalue) of the initial data set by a linear vector. Individual coordinates for higher-ranked PAs are determined in the same way as for PA1 but by constraining the direction of higher-ranked PAs to be orthogonal relative to the direction of the lower-ranked PAs. Higher-ranked PAs carried the largest possible residual variance not accounted for by lower-ranked PAs. Standard statistics can be performed on PAs resulting from a PCA, just as for any other linear variable. In the present study, all data were coded as quantitative variables (see Table 1).

PCA was run using the "Factominer" package in R[24,25].

© 2023 Routier L et al. *JAMA Network Open.*

eMethods-4. Multiple factor analysis (MFA)

The MFA is an extension of the PCA (for details of the latter, see eMethod-3) in which the initial set of variables is structured into groups and weighted in order to mitigate the influence of differences in the number of variables per group[26].

Firstly, four quantitative variable categories were defined: (i) The CRIB-II score for perinatal risk-factors; (ii) HRF, PDA, CA, HD, and NEC for postnatal morbidities; (iii) the IVH grade for brain structure risk-factors; (iv) and the richness and lability of the cEEG background, the characteristics of age-related specific features, and the negative theta central activity for brain function risk-factors (see Table 1 for the variables selected in step 1).

Secondly, the variables were reduced/centered and then weighted by dividing each of them by the square root of the largest eigenvalue of each variable category considered. Eigenvalues were determined by PCAs conducted independently for each category of variable (i.e. pools of several variables).

Lastly, a general PCA is performed on the reduced/centered/weighted variables and results in new linear orthogonal PAs to which the initial variables contribute to varying degrees[28,29]. Statistical analyses are performed on the PAs resulting from a MFA.

MFA was run using the "Factominer" package in R[24,25].

© 2023 Routier L et al. *JAMA Network Open.*

eMethods-5. Support vector machine (SVM) classification

The classification of a dataset using different methods is recommended for more reliable results[30,31]. To complete the CART, SVM classifications of favorable/adverse outcomes were performed on all the variables selected in the first step (see Table 1 for the variables selected in step 1).

A SVM classifies objects by determining the optimal separation hyperplane in the data set by maximizing the margin between the classes[32]. The hyperplane is determined such that the distance from it to the nearest data point on each side is maximized. Kernel functions were applied to take account of a potentially nonlinear best hyperplane. A kernel refers to a method that allows the application of linear classifiers to non-linear problems by mapping non-linear data into a higher-dimensional space.

SVM classifiers and other artificial intelligence methods (such as neural networks) often give good results but are "black box" approaches that prevent the use from visualizing the relative importance of and relationships between input variables (for more details, see Ambalavanan et al.[33]).

SVM was run using the "e1071" package in R[24,34].

© 2023 Routier L et al. *JAMA Network Open.*

eMethods-6. 10K-fold cross-validation and split subsets cross-validation

The performance of a supervised predictive model can be overestimated when the model is simply determined on the sample of subjects used to construct the model. Internal validation is recommended for a more accurate estimate of the model's performance[35,36].

An internal cross-validation by K-folds was performed on the CART run on the MFA and on the SVM classifications with K=10. The analysis divides the entire sample into 10 equal groups, uses 9 of the groups to create the tree, and uses the 10th group to test it. This procedure is then repeated 9 more times, always with a different 90% for building the tree[37]. Mean (range) accuracy values are reported in eResults-5.

A split subsets cross-validation was also performed. Firstly, a complementary MFA –including all the variables previously treated by the multimodal model– was conducted on a training subset composed of 74 randomly selected newborns (67% of the sample); the 35 remaining newborns (33% of the sample) composing the validation subset. The PAs coordinates of the newborns in the validation subset were determinate on the basis of the MFA conducted on the training subset. For this purpose, the newborns of the validation subset were labeled as supplementary individuals when performing the MFA which was conducted on the newborns of the training subset. Secondly, the performance of the training and the validation subsets were evaluated with AUCs.

K-fold cross-validations were run using the "caret" package in R[23,24] and MFA with the "Factominer" package[24,2]

© 2023 Routier L et al. *JAMA Network Open.*

eResults-1. Demography of the sample - Amiens and French populations comparisons

During the recruitment period (between 2013 and 2018), 318 newborns born before 28 weeks of gestational age (wGA) were admitted in the neonatal intensive care unit (NICU) of Amiens-Picardie University medical center. Among them:

- 109 newborns (34.3%) met the inclusion criteria for the present study: born at 23 to 28 of wGA; having undergone cEEG and cranial ultra-sound (cUS) examination in the first two weeks after delivery; followed until 2 years of age or died before NICU discharge;
- 84 (26.4%) newborns were not included because of the absence of a cEEG recording and/or a cranial US during the first 2 weeks after birth and for 125 infants (39.3%) because of unknown neurodevelopmental outcome at 2 years old.

The comparison between the demographic data of the 318 preterm newborns admitted to the NCIU at Amiens- Picardie University medical center to the French with the preterm newborns born at 23 to 28 of wGA from the Cohort Study EPIPAGE-2 (the national, prospective, population-based cohort study conducted in French NICU in 2011)[38] showed that the Amiens population is quite similar to the EPIPAGE-2 population in term of distribution of newborns per gestational age and in term of mortality rate (Figure [A] and [B]). The population of newborns born ≤ 28 wGA in Amiens NICU is representative of the French population of extremely preterm newborns in terms of distribution and mortality rates.

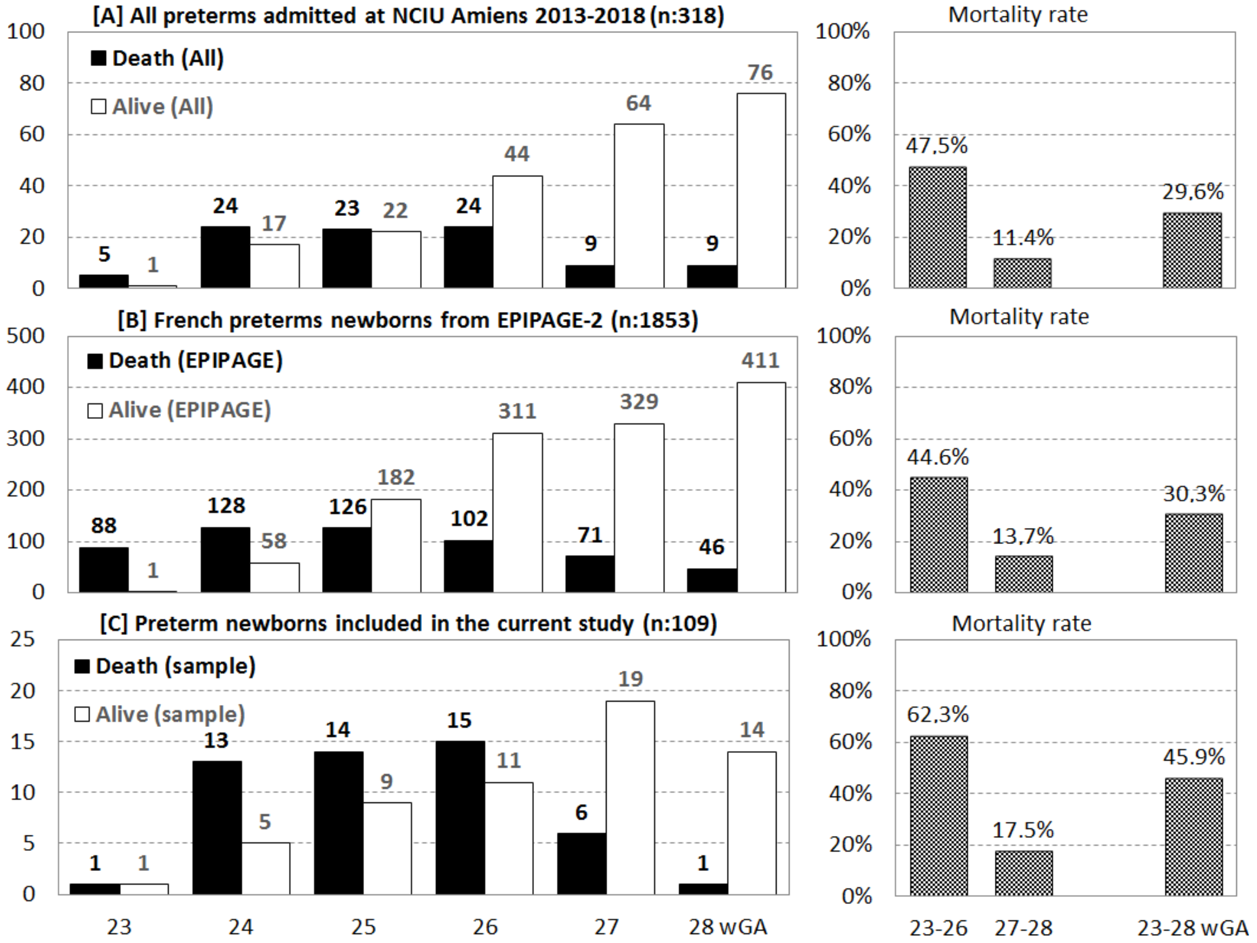

wGA: weeks of gestational age; RR: relative risk of the mortality for newborns ≤ 26 wGA compared to newborns > 26 wGA. 95%CI: 95% confidence interval.

The demographic data of the current study sample (n=109) presented some specificities. The mortality rate was higher in the study sample than in both Amiens and EPIPAGE-2 populations and was related to an overrepresentation of extremely preterm newborns born ≤ 26 wGA. Nevertheless, the relative risks between newborns at 23-26wGA and 27-28 wGA were quite similar in the Amiens population (RR=4.0) [A], EPIPAGE-2 population (RR=3.27) [B] and the current study (RR=3.56) [C].

The overrepresentation of newborns ≤ 26 wGA resulted to the retrospective and observational nature of the study. As recommended, the cEEG recordings during the neonatal period are all the more important when the child is at high neurological risk[39]. In clinical practice, because of limited access to cEEG due to technical and personnel constraints, neonatologists stratify their requests prioritizing the most at-risk newborns. This results that EEG was more frequently performed before 14 days of life in the most at-risk newborns.

© 2023 Routier L et al. *JAMA Network Open.*

eResults-2. Additional statistics: Comparisons of favorable/adverse outcome and LLST/no.LLST groups for variables of unimodal and multimodal models

Pairwise comparisons of (i) favorable vs. adverse outcome groups and (ii) LLST vs. no.LLST groups for unitary variables and the first three PAs from the PCAs and the MFA were performed using Student's t-test or the Mann-Whitney U test for ordinal and continuous variables. Type I errors for multiple comparisons were controlled by calculating the false discovery rate (FDR) (*P-value*<.05, k=22 comparisons).

**Pairwise comparisons of favorable/adverse outcome and LLST/no.LLST groups for unimodal and multimodal models**				
		Mean (SD)<br>or Median (IQR: Q1/Q3)		
**Risk-factor**		Favorable outcome group (n:52)	Adverse outcome group (n:57)	*P*-value
**Unimodal models**				
Perinatal model (CRIB-II)		10.1 (2.4)	12.9 (2.2)	<.001[a]
	PA1	-0.64 (-0.26/-1.84)	0.73 (0.00/1.56)	<.001[b]
Postnatal model (PCA1)	PA2	-0.13 (-0.46/0.51)	-0.40 (-0.60/0.61)	.50[b]
	PA3	-0.16 (-0.65/0.87)	-0.07 (-0.26/0.47)	.56[b]
Brain structure model (IVH)		1 (0-2)	3 (2-4)	<.001[b]
Brain function model (cEEG) (PCA2)	PA1<br>PA2<br>PA3	-1.20 (-0.19/-1.72)<br>-0.35 (-0.16/-0.56)<br>0.05 (-0.52/0.52)	0.36 (-0.65/2.43)<br>-0.09 (-0.35/1.17)<br>0.19 (-0.92/0.72)	<.001[b]<br><.007[b]<br>.84[b]
**Multimodal model**				
	PA1	-1.05 (1.11)	0.96 (1.11)	<.001[a]
All risk-factors (MFA)	PA2	0.03 (-0.31/0.51)	-0.10 (-0.88/0.69)	.65[b]
	PA3	-0.01 (0.75)	0.01 (0.84)	.92[a]
		Mean (SD)<br>or Median (IQR: Q1/Q3)		
**Risk-factor**		no.LLST group (n:26)	LLST group (n:24)	*P*-value
**Unimodal models**				
Perinatal model (CRIB-II)		12.7 (2.1)	13.5 (2.1)	.30[a]
	PA1	1.06 (1.27)	0.45 (1.20)	.090[a]
Postnatal model (PCA1)	PA2	-0.37 (-0.60/0.37)	-0.43 (-0.76/0.78)	.95[b]
	PA3	-0.26 (–0.45/0.16)	0.00 (-0.26/0.47)	.32[a]
Brain structure model (IVH)		2 (1/3)	4 (3/4)	.002[b]
Brain function model (cEEG) (PCA2)	PA1<br>PA2<br>PA3	0.18 (2.00)<br>-0.14 (-0.35/1.47)<br>0.04 (1.02)	1.61 (2.19)<br>-0.04 (-0.76/0.77)<br>0.03 (0.98)	.020[a]<br>.54[b]<br>.98[a]
**Multimodal model**				
	PA1	0.66 (0.93)	1.33 (1.22)	.035[a]
All risk-factors (MFA)	PA2	-0.67 (-0.09/-1.24)	0.52 (-0.31/1.27)	.001[b]
	PA3	0.10 (0.94)	0.05 (0.73)	.84[a]

LLST: limitation of life-sustaining therapies; a: Student's t-test; b: Mann-Whitney U test; All *P*-values were statistically significant after FDR correction (k=22, *P*-value<.05) expected for the PA1 (PCA2) of the brain function model (cEEG).

© 2023 Routier L et al. *JAMA Network Open.*

eResults-3. Additional information on variable contributions to the MFA

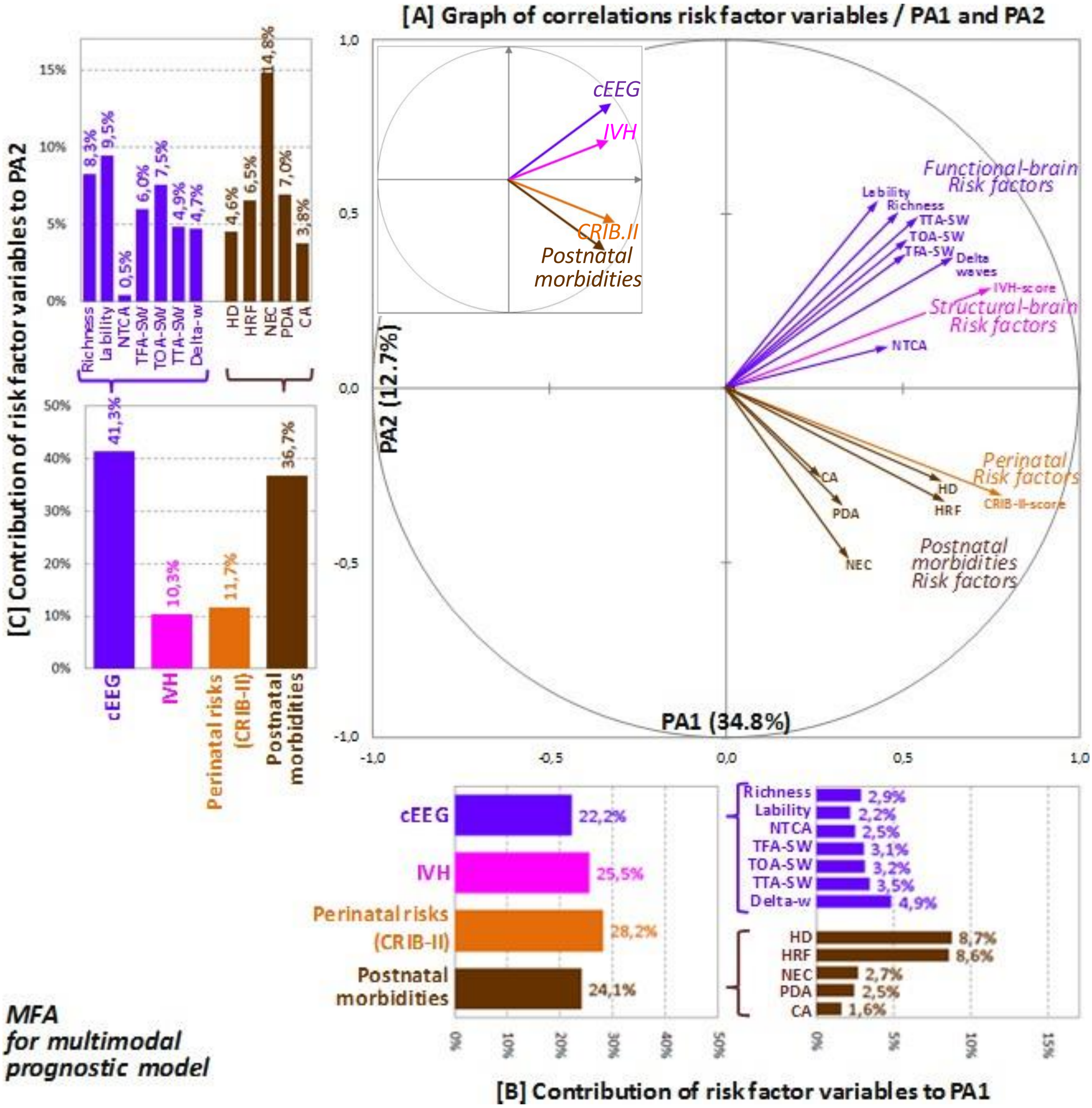

[A] Graph of the correlations between the variables selected in step 1 (for details, see Table 1) and the two first PAs from the MFA. The groups of variables defined in the MFA were the CRIB.II score for perinatal risk-factors, HD, HRF, PDA, NEC and CA for postnatal morbidities, IVH grades for brain structure risk-factors, and TOA-SW, TTA- SW, TFA-SW, delta-wave, NTCA, lability and richness of cEEG for brain function risk-factors. Length and direction of each vector determined the specific contribution of the original variable to PAs. Vectors resulting from each group of risk-factor were represented in the inset. [B] and [C] Contributions of the four risk-factor categories to PA1 and PA2, respectively. Together, PA1 and PA2 accounted for nearly 50% of the variance in the initial dataset (r^2=.48).

© 2023 Routier L et al. *JAMA Network Open.*

eResults-4. Additional MFA excluding the neonates died in LLST circumstances

To evaluate whether the limitation of life-sustaining therapies (LLST) decision could bias the model, a complementary multiple factor analysis (MFA) excluding the newborns died in LLST circumstances was performed. The AUC obtained with this complementary MFA was closed to those obtained with the analysis in the entire population. These results demonstrate that LLST decisions did not significantly impact the model and its prognostic performances for outcome in the study sample.

**Analysis**	**n**	**AUC**	**95%CI**
Full multimodal model (all newborns)	109	91.7%	[86.4-97.0]
Complementary multimodal model excluding LLST	83	88.7%	[81.4-96.1]

© 2023 Routier L et al. *JAMA Network Open.*

eResults-5. Additional statistics: SVM and 10k-fold internal cross-validation

SVM classifications were performed on the 14 variables selected in step 1: the CRIB-II score, HD, HRF, NEC, PDA, CA, IVH grade, EEG background lability, EEG background richness, Delta wave, TTA-SW, TOA-SW, TFA-SW, and NTCA.

SVM classifications gave accuracies very close to those obtained with the CART-algorithm run on the MFA. This result highlighted the good reliability of the prognosis from the multimodal risk-factor model, with classifiers operating through different mathematical tools.

The mean accuracy of 10k-fold internal cross-validation remained satisfactory (≥85% for the two classifications).

**Classification**	**Accuracy**	[95%CI]	**Specificity**	[95%CI]	**Sensitivity**	[95%CI]	**10k-fold mean accuracy**	Range
SVM	89.0%	[81.6-94.2]	93.0%	[83.0-98.1]	84.6%	[71.9-93.1]	88.5%	[80.0-100]
$CART_{(on\ MFA)}$	87.2%	[79.4-92.8]	88.0%	[75.6-95.5]	86.4%	[75.0-94.0]	85.0%	[70.0-100]

SVM: support vector machine; CART: classification and regression tree; MFA: multiple factor analysis; Range: minimum and maximum accuracy values of the 10k-fold internal cross validation.

Although the SVM classification performed better than the CART run on the MFA, the latter was preferred to because it enables the use to know which variables contributed to the model –and to what extent–, which is crucial for a clinical application.

© 2023 Routier L et al. *JAMA Network Open.*

eResults-6. Additional statistics: split subsets cross-validation

The AUCs of the complementary MFA for the training subset (74 randomly selected newborns: 67% of the sample) and the validation subset (35 newborns: 33% of the sample) were very closed, and both similar to the AUC obtained with the full multimodal model.

**Analysis**	**n**	**AUC**	**95%CI**
Model building subset (67% of the sample)	74	91.1%	[87.5-100]
Model validation subset (33% of the sample)	35	94.3%	[73.6-98.3]
Full multimodal model (100% of the sample)	109	91.7%	[86.4-97.0]

© 2023 Routier L et al. *JAMA Network Open.*

eReferences

1. Parry G, Tucker J, Tarnow-Mordi W, UK Neonatal Staffing Study Collaborative Group. CRIB II: an update of the clinical risk index for babies score. *Lancet*. 2003;361(9371):1789-1791. doi:10.1016/S0140-6736(03)13397-1

2. Conde-Agudelo A, Romero R, Jung EJ, Garcia Sánchez ÁJ. Management of clinical chorioamnionitis: an evidence-based approach. *Am J Obstet Gynecol*. 2020;223(6):848-869. doi:10.1016/j.ajog.2020.09.044

3. Newton ER. Preterm Labor, Preterm Premature Rupture of Membranes, and Chorioamnionitis. *Clin Perinatol*. 2005;32(3):571-600. doi:10.1016/j.clp.2005.05.001

4. Bell MJ, Ternberg JL, Feigin RD, et al. Neonatal necrotizing enterocolitis. Therapeutic decisions based upon clinical staging. *Ann Surg*. 1978;187(1):1-7. doi:10.1097/00000658-197801000-00001

5. Richmond S, Wyllie J. European Resuscitation Council Guidelines for Resuscitation 2010: Section 7. Resuscitation of babies at birth. *Resuscitation*. 2010;81(10):1389-1399. doi:10.1016/j.resuscitation.2010.08.018

6. Papile LA, Burstein J, Burstein R, Koffler H. Incidence and evolution of subependymal and intraventricular hemorrhage: A study of infants with birth weights less than 1,500 gm. *J Pediatr*. 1978;92(4):529-534. doi:10.1016/S0022-3476(78)80282-0

7. Mohammad K, Scott JN, Leijser LM, et al. Consensus Approach for Standardizing the Screening and Classification of Preterm Brain Injury Diagnosed With Cranial Ultrasound: A Canadian Perspective. *Front Pediatr.* 2021;9. doi: 10.3389/fped.2021.618236

8. de Vries LS, Eken P, Dubowitz LMS. The spectrum of leukomalacia using cranial ultrasound. *Behav Brain Res.* 1992;49(1):1-6. doi:10.1016/S0166-4328(05)80189-5

9. Bourel-Ponchel E, Gueden S, Hasaerts D, et al. Normal EEG during the neonatal period: maturational aspects from premature to full-term newborns. *Neurophysiol Clin*. 2021;51(1):61-88. doi:10.1016/j.neucli.2020.10.004

10. André M, Lamblin MD, d'Allest AM, et al. Electroencephalography in premature and full-term infants. Developmental features and glossary *Neurophysiol Clin*. 2010;40(2):59-124. doi:10.1016/j.neucli.2010.02.002

11. Lamblin MD, André M, Challamel MJ, et al. Électroencéphalographie du nouveau-né prématuré et à terme. Aspects maturatifs et glossaire. *Neurophysiol Clin*. 1999;29(2):123-219. doi:10.1016/S0987-7053(99)80051-3

12. Wallois F, Routier L, Heberlé C, Mahmoudzadeh M, Bourel-Ponchel E, Moghimi S. Back to basics: the neuronal substrates and mechanisms that underlie the electroencephalogram in premature neonates. *Neurophysiol Clin*. 2021;51(1):5-33. doi:10.1016/j.neucli.2020.10.006

13. Tich SNT, d'Allest AM, Villepin AT de, et al. Pathological features of neonatal EEG in preterm babies born before 30 weeks of gestationnal age. *Neurophysiol Clin*. 2007;5(37):325-370. doi:10.1016/j.neucli.2007.10.001

14. Watanabe K, Hayakawa F, Okumura A. Neonatal EEG: a powerful tool in the assessment of brain damage in preterm infants. *Brain Dev*. 1999;Sep;21(6):361–72. doi: 10.1016/s0387-7604(99)00034-0

15. Hayashi-Kurahashi N, Kidokoro H, Kubota T, et al. EEG for Predicting Early Neurodevelopment in Preterm Infants: An Observational Cohort Study. *Pediatrics.* 2012;130(4):e891-e897. doi:10.1542/peds.2012-1115

16. Marret S, Parain D, Jeannot E, Eurin D, Fessard C. Positive rolandic sharp waves in the EEG of the premature newborn: a five year prospective study. *Arch Dis Child*. 1992;67(7):948-951. doi: 10.1136/adc.67.7.948

17. Marret S, Parain D, Ménard JF, Blanc T, Devaux AM, Ensel P. Prognostic value of neonatal electroencephalography in premature newborns less than 33 weeks of gestational age. *Electroencephalogr Clin Neurophysiol.* 1997;102(3):178–85. doi: 10.1016/s0013-4694(96)95655-6

18. Baud O, Nedelcoux H, Boithias C, Delaveaucoupet J, Dehan M. TThe early diagnosis of periventricular leukomalacia in premature infants with positive rolandic sharp waves on serial electroencephalography. *J Pediatr.* 1998;132(5):813-817. doi:10.1016/S0022-3476(98)70309-9

19. Pressler RM, Cilio MR, Mizrahi EM, et al. The ILAE classification of seizures and the epilepsies: Modification for seizures in the neonate. Position paper by the ILAE Task Force on Neonatal Seizures. *Epilepsia*. 2021;62(3):615-628. doi:10.1111/epi.16815

20. Breiman L, Friedman JH, Olshen RA, Stone CJ. *Classification And Regression Trees*. Routledge; 2017. doi:10.1201/9781315139470

21. Laptook AR, Shankaran S, Ambalavanan N, et al. Outcome of term infants using apgar scores at 10 minutes following hypoxic-ischemic encephalopathy. *Pediatrics*. 2009;124(6):1619-1626. doi:10.1542/peds.2009-0934

22. Hothorn T, Zeileis A. Partykit: A Modular Toolkit for Recursive Partytioning in R. *J Mach Learn Res*. 2015;16(118):3905-3909. doi:10.5555/2789272.2912120

23. Kuhn M. Building Predictive Models in R Using the caret Package. *J Stat Softw*. 2008;28:1-26. doi:10.18637/jss.v028.i05

24. R Core Team. R: A Language and Environment for Statistical Computing. R Foundation for Statistical Computing. https://www.R-project.org/. 2013.

25. Lê S, Josse J, Husson F. FactoMineR: An R Package for Multivariate Analysis. *J Stat Softw*. 2008;25:1-18. doi:10.18637/jss.v025.i01

26. Escofier B, Pagès J. Multiple factor analysis (AFMULT package). *Comput Stat Data Anal.* 1994;18(1):121-140. doi:10.1016/0167-9473(94)90135-X

27. Wyckoff MH, Aziz K, Escobedo MB, et al. Part 13: Neonatal Resuscitation: 2015 American Heart Association Guidelines Update for Cardiopulmonary Resuscitation and Emergency Cardiovascular Care. *Circulation*. 2015;132(18 Suppl 2):S543-560. doi:10.1161/CIR.0000000000000267

28. Kostov B, Bécue-Bertaut M, Husson F. Multiple Factor Analysis for Contingency Tables in the FactoMineR Package. *The R Journal*. 2013;5(1):29-38.

29. Abdi H, Williams LJ, Valentin D. Multiple factor analysis: principal component analysis for multitable and multiblock data sets. *WIREs Comput Stat*. 2013;5(2):149-179. doi:10.1002/wics.1246

30. d'Huy J. Polyphemus (Aa. Th. 1137): A phylogenetic reconstruction of a prehistoric tale. *Nouvelle Mythologie Comparée/New Comparative Mythology*. 2013;1(1).

31. Kiang MY. A comparative assessment of classification methods. *Decision Support Systems*. 2003;35(4):441-454. doi:10.1016/S0167-9236(02)00110-0

32. Cortes C, Vapnik V. Support-vector networks. *Mach Learn*. 1995;20(3):273-297. doi:10.1007/BF00994018

33. Ambalavanan N, Nelson KG, Alexander G, Johnson SE, Biasini F, Carlo WA. Prediction of neurologic morbidity in extremely low birth weight infants. *J Perinatol*. 2000;20(8 Pt 1):496-503. doi:10.1038/sj.jp.7200419

34. Meyer D, Wien TU. Support Vector Machines. The Interface to libsvm in package. 2015;e1071

35. Crilly CJ, Haneuse S, Litt JS. Predicting the outcomes of preterm neonates beyond the neonatal intensive care unit: What are we missing? *Pediatr Res*. 2021;89(3):426-445. doi:10.1038/s41390-020-0968-5

36. Steyerberg EW, Harrell FE, Borsboom GJ, Eijkemans MJ, Vergouwe Y, Habbema JD. Internal validation of predictive models: efficiency of some procedures for logistic regression analysis. *J Clin Epidemiol*. 2001;54(8):774-781. doi:10.1016/s0895-4356(01)00341-9

37. McKee LA, Fabres J, Howard G, Peralta-Carcelen M, Carlo WA, Ambalavanan N. PaCO2 and neurodevelopment in extremely low birth weight infants. *J Pediatr*. 2009;155(2):217-21.e1. doi:10.1016/j.jpeds.2009.02.024

38. Ancel PY, Goffinet F, Kuhn P, et al. Survival and Morbidity of Preterm Children Born at 22 Through 34 Weeks' Gestati on in France in 2011: Results of the EPIPAGE-2 Cohort Study. *JAMA Pediatr*. 2015;169(3):230. doi:10.1001/jamapediatrics.2014.3351

39. Malfilâtre G, Mony L, Hasaerts D, Vignolo-Diard P, Lamblin MD, Bourel-Ponchel E. Technical recommendations and interpretation guidelines for electroencephalography for premature and full-term newborns. *Neurophysiol Clin*. 2021;51(1):35-60. doi:10.1016/j.neucli.2020.10.005

© 2023 Routier L et al. *JAMA Network Open.*
