# Supplementary material for: Predicting the Neurodevelopmental Outcome in Extremely Preterm Newborns Using a Multimodal Prognostic Model Including Brain Function Information
Source: JAMA Netw Open. 2023 Mar 8;6(3):e231590. doi: 10.1001/jamanetworkopen.2023.1590 (PMC9996404; doi:10.1001/jamanetworkopen.2023.1590)
Supplement: Supplement 2. — Data Sharing Statement [file jamanetwopen-e231590-s002.pdf]

Data Sharing Statement

Routier. Predicting the Neurodevelopmental Outcome in Extremely Preterm Newborns Using a Multimodal Prognostic Model Including Brain Function Information. *JAMA Netw Open*. Published March 08, 2023. doi:10.1001/jamanetworkopen.2023.1590

Data
Data available: No
